# Cytidine Triphosphate Synthase Four From *Arabidopsis thaliana* Attenuates Drought Stress Effects

**DOI:** 10.3389/fpls.2022.842156

**Published:** 2022-03-10

**Authors:** Moritz Krämer, Eva Dörfer, Daniel Hickl, Leo Bellin, Vanessa Scherer, Torsten Möhlmann

**Affiliations:** Plant Physiology, Faculty of Biology, University of Kaiserslautern, Kaiserslautern, Germany

**Keywords:** Arabidopsis, nucleotides, *de novo* synthesis, CTP-synthase, drought stress, salt stress

## Abstract

Cytidine triphosphate synthase (CTPS) catalyzes the final step in pyrimidine *de novo* synthesis. In Arabidopsis, this protein family consists of five members (CTPS1–5), and all of them localize to the cytosol. Specifically, *CTPS4* showed a massive upregulation of transcript levels during abiotic stress, in line with increased staining of *CTPS4* promoter:GUS lines in hypocotyl, root and to lesser extend leaf tissues. In a setup to study progressive drought stress, *CTPS4* knockout mutants accumulated less fresh and dry weight at days 5–7 and showed impaired ability to recover from this stress after 3 days of rewatering. Surprisingly, a thorough physiological characterization of corresponding plants only revealed alterations in assimilation and accumulation of soluble sugars including those related to drought stress in the mutant. Bimolecular fluorescence complementation (BiFC) studies indicated the interaction of CTPS4 with other isoforms, possibly affecting cytoophidia (filaments formed by CTPS formation. Although the function of these structures has not been thoroughly investigated in plants, altered enzyme activity and effects on cell structure are reported in other organisms. CTPS activity is required for cell cycle progression and growth. Furthermore, drought can lead to the accumulation of reactive oxygen species (ROS) and by this, to DNA damage. We hypothesize that effects on the cell cycle or DNA repair might be relevant for the observed impaired reduced drought stress tolerance of *CTPS4* mutants.

## Introduction

Due to climate change, plants are facing ever-increasing challenges. Especially drought and salt stress lead to large losses in plant growth and productivity. Above all, an adequate water supply is essential for regulated growth, whereas periods of drought lead to major changes in metabolism as it represents a stress situation for them (Bray, 2004). Thereby, plants naturally sense and respond to water stress by activating specific signaling pathways leading to physiological and developmental adaptations. The detection of drought basically takes place through two different signaling pathways.

The abscisic acid (ABA)-dependent pathway is activated in the early acclimation phase (Verslues and Bray, 2006; Singh and Laxmi, 2015). Here, ABA receptors inhibit the activity of protein phosphatase 2C (PP2C), a negative regulator of the pathway due to dephosphorylation of three SNF1-related protein kinases (*SnRK2.2*; *SnRK2.3*; *SnRK2.6*) (Ben-Ari, 2012; Singh and Laxmi, 2015; Song et al., 2015). Thus, inhibition of PP2C releases the inhibitory effect on SnRK2s. Active SnRK2s phosphorylate the ABA-responsive element binding protein and its binding factor AREB, and by this activate many ABA-dependent genetic adaptations to drought (Kobayashi et al., 2005; Singh and Laxmi, 2015). Another major effect of ABA in drought response is related to the control of stomatal aperture (Munemasa et al., 2015).

In the ABA-independent pathway, the dehydration-responsive element (DRE) binding protein isoform 2A (*DREB2A*) is expressed upon drought stress and regulates the expression of various downstream genes by binding to the DRE, and to different *cis*-acting elements (CRTs) (Sakuma et al., 2006; Singh and Laxmi, 2015). To do so, DREB2A requires post-translational activation because of an intrinsic negative regulatory sequence (Sakuma et al., 2006), whereas inactive DREB2A is degraded in proteasomes (Singh and Laxmi, 2015).

Both signaling pathways most likely possess mechanisms by which they can interact and influence each other. However, from the underlying regulation of each pathway itself and the pathways they regulate, they should be considered separate largely independent pathways (Singh and Laxmi, 2015).

Drought stress leads to decreased water potential, increased levels of reactive oxygen species (ROS), and consequently to cell damage (Mukarram et al., 2021). A typical plant response is the accumulation of solutes like the monosaccharides glucose and fructose, those of the raffinose family oligosaccharides, and the amino acid proline (Fabregas and Fernie, 2019).

Although it is known that pyrimidine nucleotides are important components of a variety of different biological processes in all living organisms, the role of nucleotide metabolism in adaptation to drought stress in plants has been elusive. As nucleotides are important building blocks of nucleic acids, they are involved in the storage and dissemination of genetic information. In addition, they act as energy transmitters and as precursors for the synthesis of primary metabolic products such as sucrose, polysaccharides, and phospholipids (Moffatt and Ashihara, 2002; Zrenner et al., 2006).

Nucleotides can either be synthesized *de novo* or by salvage from nucleobases and nucleosides, representing intermediates in catabolism. Plants exhibit a unique organization of pyrimidine *de novo* synthesis as the first committed and highly regulated step catalyzed by aspartate transcarbamoylase localizes to chloroplasts, whereas the remaining reactions occur outside this organelle (Chen and Slocum, 2008; Witz et al., 2012; Bellin et al., 2021a). The final and rate limiting step in nucleotide *de novo* biosynthesis is the formation of cytidine triphosphate (CTP) catalyzed by CTP synthase (CTPS), a highly conserved enzyme in all prokaryotes and eukaryotes investigated (Long and Pardee, 1967; Wadskov-Hansen et al., 2001; Evans and Guy, 2004; Daumann et al., 2018).

All cytosine-based metabolites are initially formed by CTPS rendering this enzyme activity essential for all organisms, even parasites with a streamlined genome (Danchin and Marliere, 2020). Because of this central regulatory role, accurate regulation of CTPS enzyme activity is critical. In addition to post-translational modifications, enzymatic activity is regulated by allosteric factors such as GTP and *via* feedback inhibition by CTP (Levitzki and Koshland, 1972; Chang and Carman, 2008). Recently, filamentous CTPS structures named cytoophidia were identified and meanwhile shown to exist in all kingdoms of life (Ingerson-Mahar et al., 2010; Liu, 2010; Noree et al., 2010; Lynch et al., 2017; Zhou et al., 2020). Compartmentation of CTPS in the form of cytoophidia is at the crossroads of metabolic control, as these structures can represent the active or inactive enzyme form, and the activity can even be altered within cytoophidia very fast (Barry et al., 2014; Lynch et al., 2017; Lynch and Kollman, 2020). Although this filament formation has been studied in more detail in various bacteria and eukaryotes including yeast, drosophila, and humans, the physiological relevance in plants is unclear. Although non-plant species studied so far exhibit a maximum of two CTPS isoforms, Arabidopsis possesses five isoforms. Studies revealed that only the CTPS 3-5 formed filaments, whereas CTPS1 and 2 each appeared diffusely localized to the cytoplasm (Daumann et al., 2018; Alamdari et al., 2021).

With respect to physiological function, CTPS2 was characterized as essential for complete embryo development (Hickl et al., 2021). Knockdown mutants for *CTPS2* revealed lower amounts of plastid DNA and RNA accompanied by reduced chlorophyll levels and impaired photosynthesis, especially in seedlings (Bellin et al., 2021b). T-DNA insertion lines leading to loss of function for *CTPS1*, *3*, *4*, and *5* showed no apparent phenotypic differences to the wild type (WT) under standard growth conditions (Daumann et al., 2018). To investigate whether CTPS isoforms exhibit individual roles under non-standard growth, the GENEVESTIGATOR expression database was screened. In fact, *CTPS4* showed a significantly increased expression under drought and salt stress (Hruz et al., 2008). Therefore, in the course of this work, we tested a possible contribution of CTPS4 in drought stress attenuation by expression analysis, subjecting knockout mutants to progressive drought, analysis of promoter activity, and interaction studies between CTPS isoforms by bimolecular fluorescence complementation (BiFC).

## Materials and Methods

### Plant Growth

For DNA isolation, tissue collection and phenotypic inspection, WT, and transgenic *Arabidopsis thaliana* (L.) Heynh. plants [ecotype Columbia (Col-0)] including single knockout lines for *CTPS4* (*ctps4-1*, SALK_020074C and *ctps4-2*, SALK_127028C) (Daumann et al., 2018) were used throughout. Plants were grown on standardized ED73 soil (Einheitserde and Humuswerke Patzer) or on agar plates under a regime of 10 h light (120 μmol quanta m^–2^s^–1^) and 14 h darkness at 22°C and 60% humidity. For illumination, LED lights (Valoya NS1, Valoya, Finland) were used.

Drought stress experiments on soil were performed as described in Harb et al. (2010) with slight modifications. In brief, plants were grown for 21 days under standard conditions. Afterward, plants were not watered for up to 9 to 10 days. For recovery experiments from drought stress, plants were watered again after 3, 5, and 7 days of drought for 3 days each. The experiment was run three times in total, once including both *CTPS4* mutants, twice including *ctps4-1* alone (not shown). The observed difference in the drought response between WT and mutants was highly similar in all three runs.

To apply salt stress, the method was used as described in Seok et al. (2017) with slight modifications. Here, plants were grown again for 21 days under standard conditions before watering the plants with 100 mM NaCl for 10 days. For determination of the plant dry weight, the harvested plants were collected in 5 ml reaction tubes without a lid and dried for 48 h at 65°C.

For growth experiments on half strength Murashige and Skoog (MS) agar (without sucrose), seeds were surface sterilized and incubated for 24 h in the dark at 4°C for imbibition (Weigel and Glazebrook, 2002). For drought stress experiments, the agar plates were supplemented with PEG 6000 to create a negative water potential of −0.5 MPa. Salt stress experiments were performed with 100 mM NaCl added to the agar plates.

Furthermore, drought experiments were performed on hydroponic cultures. For that, seeds and 1/2 MS medium were prepared according to the standard protocols (Conn et al., 2013). To simulate drought stress, the medium was supplemented with PEG 6000 to create a negative water potential of −0.5 MPa.

### Analysis on Abscisic Acid-Dependent or -Independent Pathway

To determine the ABA-effect, 15 leaf discs (0.5 cm) from three different non-transgenic and transgenic plants were stamped out and soaked for 2 h in liquid 1/2 MS medium containing either 0 μM, 10 μM, or 50 μM ABA. For normalization, untreated leaf (0 μM) discs were incubated in 1/2 MS medium without ABA. Five leaf discs were combined into one biological replicate and subsequently used in the gene expression analysis (*n* = 3).

### Gene Expression Analysis

Harvested leaf material was frozen in liquid nitrogen and then homogenized for the isolation of total RNA with the Nucleospin RNA Plant Kit (Macherey-Nagel, Düren, Germany) according to the manufacturer’s instructions. The purity and concentration of the RNA were quantified by using a NanoDrop spectrophotometer (Thermo Fisher Scientific). RNA was used for cDNA synthesis with the qScript cDNA Synthesis Kit (Quantabio, United States). The Quantabio SYBR Green Quantification Kit (Quantabio) on the PFX96 system (Bio-Rad, Hercules, CA, United States) was used for quantitative reverse transcription-PCR (qRT-PCR) with specific Primers (Supplementary Table 1). For normalization, *Actin2* (At3g18780) was used as a reference gene.

### Generation of *proCTPS4*:GUS Constructs and Histochemical Staining

For the analysis of *CTPS4* expression by histochemical staining for GUS activity, *proCTPS4*:GUS lines were created by using Gateway Cloning in the pBGWFS7.0 vector as described in Hickl et al. (2021). A DNA fragment 1,829 bp upstream of the CTPS4 coding region was amplified for this purpose with primers listed in Supplementary Table 1. The resulting construct was introduced into Agrobacterium strain GV3101. The following transformation of Arabidopsis was conducted according to the floral dip method (Narusaka et al., 2010). For GUS staining, transgenic plants were collected in six-well plates. The staining was performed according to the standard protocols (Weigel and Glazebrook, 2002).

### Pulse-Amplitude Modulation Fluorometry Measurements

For *in vivo* chlorophyll fluorescence measurements, a MINI-IMAGING pulse-amplitude modulation (PAM) fluorometer (Walz Instruments, Effeltrich, Germany) was used. Induction curve assays were performed on intact plants, which were 20 min dark-adapted on standard protocols (Schreiber et al., 2007).

### Quantification of Soluble Sugars

For the ethanolic extraction, 100 mg of leaf tissue was mortared in liquid nitrogen and stored until use. The crushed material was extracted two times with 1 ml of 80% (v/v) ethanol at 80°C for 10 min. Combined extracts were evaporated in a Vacufuge concentrator (Eppendorf), and pellets were dissolved in deionized water. The determination of the sugar content in the ethanolic extracted samples was carried out by means of ion chromatography (IC). For this purpose, an 871-compact IC device (Metrohm, Herisau, Switzerland) equipped with a Metrosep Carb 2-250/4.0 column was used. The mobile phase was 0.1 M NaOH, 10 mM sodium acetate. Quantification was performed by pulsed amperometric detection.

### Gas Exchange Measurements

Gas exchange-related parameters were analyzed with a GFS-3000 system (Heinz Walz, Effeltrich, Germany). Measurements were performed with three plants, and each plant was measured three times (technical replicates). Individual plants were placed in a whole-plant gas exchange cuvette and CO2-assimilation rate, respiration, leaf CO2 concentration, and stomatal conductance were recorded. Temperature, humidity, and CO2 concentrations of the cuvette were set to the conditions plants were grown at. Light respiration was measured at PAR 125 and dark respiration at PAR 0 over a time of 1 min for each plant. Each plant was measured three times with 30-s intervals between measurements to allow leaves to return to the stabilized value.

### Bimolecular Fluorescence Complementation for Interaction Studies

Cloning for BiFC interaction studies was carried out with the full-length CTPS1-4 based on existing constructs (Daumann et al., 2018). After initial gateway cloning into pDONR^®^ *CTPS* genes were introduced into pUBC-cYFP, pUBC-nYFP, pUBN-cYFP, and pUBN-nYFP vectors (Grefen et al., 2010), resulting in localization of yellow fluorescent protein (YFP) halves (nYFP or cYFP) to the N- or C-terminus of CTPSX. These constructs were then transformed into *Agrobacterium tumefaciens* strain GV3101.

Transient expression of CTPS1-4 fused to YFP was performed as detailed in Walter et al. (2004). Therefore, 6-week-old *Nicotiana benthamiana* leaves were infiltrated through the lower epidermis. After 4 to 5 days, leaves were analyzed for the presence of fluorescence signals with a Leica TCS SP5II microscope (514 nm excitation and 525–582 nm detection of emission through an HCX PL APO 63 × 1.2 W water immersion objective). Chlorophyll autofluorescence was detected with 514 nm excitation and a 651–704 nm emission wavelength. Sequences of gene-specific primers, which were used, are provided in Supplementary Table 1.

## Results

### *CTPS4* Expression Is Highly Upregulated Upon Drought and Salt Stress

The CTPS catalyzes an essential reaction by providing CTP as a precursor for RNA and DNA synthesis and lipid synthesis in addition. However, it is not clear why Arabidopsis harbors five isoforms of this enzyme. Until now, only CTPS2 was analyzed in more detail with respect to its physiological function (Alamdari et al., 2021; Bellin et al., 2021b; Hickl et al., 2021).

To explore the putative functions of the other CTPS isoforms, the GENEVESTIGATOR expression data repository was screened. It was apparent that *CTPS4* (At4g20320) expression was strongly increased upon drought and salt stress (Hruz et al., 2008; Zhang et al., 2008).

**FIGURE 1:**
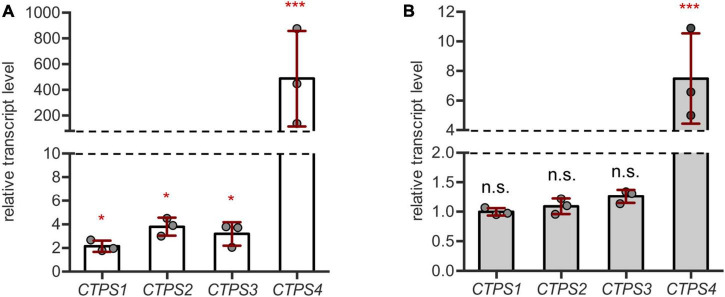
*CTPS4* expression is highly upregulated upon drought and salt stress. **(A)** Relative transcript levels of *CTPS1-4* in 31-day-old wild type (WT) plants after 10 days of drought stress. Expression was compared to watered plants (control, set to 1), after normalization to *Actin2*. **(B)** Analysis of relative transcript levels of *CTPS1-4* in 31-day-old WT plants which were grown on soil for 21 days under standard conditions and then watered with 150 mM NaCl to induce salt stress for 10 days. Expression was compared to control plants without NaCl treatment (set to 1), after normalization to *Actin2*. Plotted are the means of three biological replicates ± SD. For statistical analysis, one-way ANOVA was performed followed by Dunnett’s multiple comparison test (*p < 0.05, ***p < 0.001, n.s. no significance).

First, we aimed to corroborate the genome wide expression data by qPCR on our own drought and salt stress-inducing experimental setups. For this, plants were first grown for 21 days under short-day conditions in the soil in 60 mm pots with a regular water supply. Subsequently, pots were soaked dry with paper towels and grown for 10 more days without irrigation. After this time, whole rosette material was harvested and used for cDNA preparation. *CTPS1-4* transcript levels were found to increase compared to regularly watered controls. However, while *CTPS1-3* showed only a relatively small increase in transcript levels up to 4-fold, transcripts of *CTPS4* were increased nearly 500-fold (Figure 1A). *CTPS5* was not tested, as its expression is mainly restricted to pollen (Daumann et al., 2018).

For salt stress experiments, plants were grown on soil for 21 days and then either watered with NaCl (150 mM) containing water or fresh water as a control. Following 10 days of growth, the transcript levels of *CTPS1-4* were analyzed by qPCR. Here, only *CTPS4* showed a 7.5-fold increase in transcript level, whereas the other CTPS isoforms were unaffected (Figure 1B). Although the transcript level of *CTPS4* was greatly increased under both drought (Figure 1A) and salt stress (Figure 1B) compared to control conditions, the overall expression was still lower compared to *CTPS2* and *3* and matched that of *CTPS1* (Supplementary Figures 1A,B).

### Histochemical Staining of *CTPS4* Promoter GUS (*proCTPS4:GUS*) Lines Reveals Tissue-Specific Expression in the Hypocotyl and Roots With Increasing Intensity Upon Drought and Salt Stress

**FIGURE 2:**
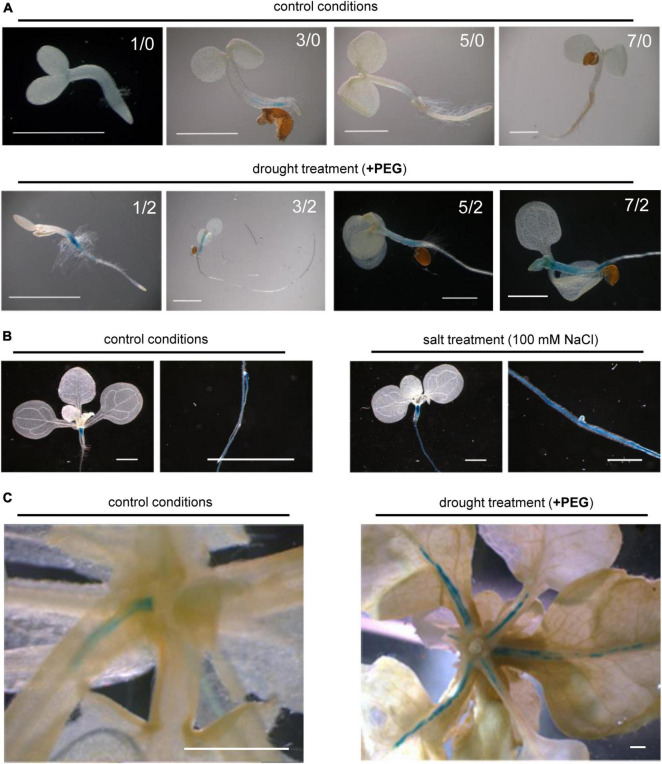
Histochemical staining of *proCTPS4:*GUS seedlings treated with drought or salt stress. **(A)** *proCTPS4:*GUS staining of plants grown on 1/2 MS medium with or without PEG6000 supplementation to induce drought stress (days grown before/after the onset of stress). **(B)** *proCTPS4:*GUS staining of plants on MS agar for 12 days with subsequent transfer to plates supplemented with 100 mM NaCl for another 2 days to induce salt stress. **(C)** *proCTPS4:*GUS staining of 26-day-old plants grown in hydroponic culture before transfer to PEG 6000 containing medium for another 5 days. Scale bar = 1 mm.

To analyze the tissue-specific expression patterns of *CTPS4* in detail, 1,829 bp upstream of the respective start codon was fused to the GUS open reading frame. After an initial screening of several lines exhibiting identical staining patterns, one line was used for further studies. Typical examples of GUS staining patterns are shown in Figure 2. Expression of *proCTPS4:GUS* was investigated in unstressed seedlings and 12-day-old plants and in parallel in drought and salt stressed plants of the same age. Thereby control conditions showed staining in the hypocotyl, between the root crown and cotyledons. While a decrease in coloration could be observed during the first 7 days under standard conditions (Figure 2A), 12-day-old plants again showed an intense coloration of the hypocotyl and roots (Figures 2B,C).

In order to analyze whether drought treatment influences *proCTPS4:*GUS lines, transgenic plants were grown on 1/2-MS medium for 1, 3, 5, and 7 days and then transferred for 2 days on 1/2-MS plates containing PEG 6000 before staining. The induction of drought stress thereby resulted in an increased coloration of the hypocotyl compared to control conditions, which remained consistently high over the first 7 days of development. In addition, the coloration of the roots was observed over the first 7 days (Figure 2A).

To induce salt stress, transgenic plants were grown on 1/2 MS medium with 100 mM NaCl for 12 days before staining. Comparable to the observations under drought stress, the application of salt stress showed increased coloration in the hypocotyl and central cylinder of *proCTPS4:*GUS plants compared to control conditions (Figure 2B).

In further experiments, it was also in our interest to see whether comparable observations can be made in older plants during vegetative growth under drought stress. Therefore, plants were first grown in liquid cultures under standard conditions for 21 days before being subjected to drought stress for 5 days. The comparison with the control conditions showed that especially the expression in the vascular tissues of rosette leaf petioles and leaf base was markedly increased after the application of drought stress (Figure 2C).

### Loss of *CTPS4* Results in Reduced Tolerance of Arabidopsis to Drought Stress

**FIGURE 3:**
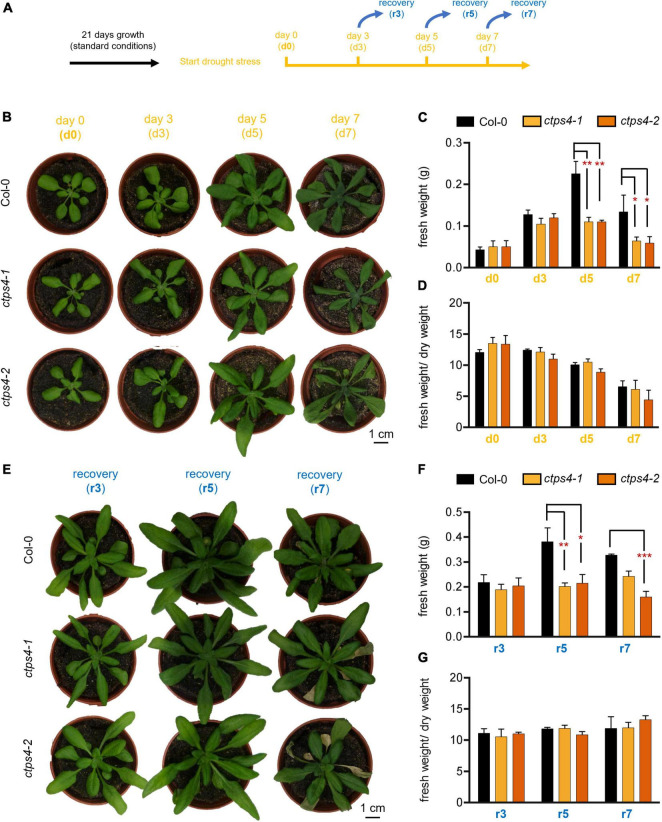
Effects of progressive drought stress on *CTPS4* knockout plants. **(A)** Scheme of experimental setup to apply drought stress. Plants were grown under a 10 h light/14 h dark regime for 21 days and watered regularly. To induce drought stress, watering of plants was stopped for 0, 3, 5, and 7 days (d0, d3, d5, and d7). **(B)** Representative plants used for the determination of **(C)** fresh weight and included in the calculation of **(D)** fresh weight to dry weight ratio. To check recovery, the plants were subsequently watered again for 3 days after **(E)** 3, 5, and 7 days (r3, r5, and r7) and further used to determine **(F)** fresh weight and to calculate **(G)** fresh weight-to-dry weight ratio. Plotted are the means of *n* = 3 biological replicates ± SD. For statistical analysis, one-way ANOVA was performed followed by Dunnett’s multiple comparison test (*p < 0.05, **p < 0.01, ***p < 0.001). Scale bar in **(A)** and **(E)** = 1 cm.

In subsequent experiments, two knockout (T-DNA insertion) lines (*ctps4-1* and *ctps4-2*) were used, previously described in Daumann et al. (2018). As drought treatment showed a more prominent *CTPS4* expression response compared to salt stress, we focus on this stress condition for the remainder of the work. To apply drought stress, WT and knockout plants were first grown for 21 days on soil in 60-mm size pots filled with standardized soil [Einheitserde ED73 and sand (10%)] watered regularly before the water supply was subsequently stopped (Figure 3A). After 0, 3, 5, and 7 days, the plants were documented (Figures 3B–D). During this progressive drought (pDR) phase, pot weight declined to 54% at day 7, accompanied by a massive increase in the expression of the drought-dependent genes *DREB2A* and *SnRK2.6* (Supplementary Figure 3).

While no differences between the WT and the mutants could be seen after 3 days of drought stress, differences in fresh weight (FW; Figures 3B,C) and dry weight (DW; Supplementary Figure 2A) of the plants appeared after 5 and 7 days. Thereby, FW/DW ratios were not different in mutants compared to WT corroborating an actual loss of biomass in the mutants and not changes in relative water content (Figure 3D).

To gain further insights into the ability of *CTPS4* knockout plants to recover from a drought period of 3, 5, and 7 days, plants of the WT and the knockout lines were rewatered again for 3 days before the plants were documented (Figures 3A,E–G). WT and mutant plants recovered equally well from 3 days of drought, after 5 and 7 days of drought; however, mutants performed worse compared to WT (Figure 3E). A comparison of the biomass revealed similar losses in FW and DW after 5 and 7 days of drought stress (Figure 3F; Supplementary Figures 2B,C). The number of wilted leaves after 7 days of drought increased from 0.7 in WT to 4 and 4.3 in *ctps4-1* and *2*, respectively (Supplementary Figures 2C,D).

To envision the water loss, pot weights (after removal of plants) were determined. Seven days without watering reduced pot weights by 46% (Supplementary Figure 3). Gene expression analysis of drought markers *DREB2A* and *SnRK2.6* after 7 days without watering led to a 1,800-fold and 9-fold increase, respectively, in WT. There were no statistical differences in *DREB2A* or *SnRK2.6* expression between WT and c*tps4-1/2* mutants (Supplementary Figures 3B,C).

### *CTPS4* Knockout Mutants Show No Impaired Photosynthetic Efficiency but Altered CO_2_ Assimilation Under Drought Stress

**FIGURE 4:**
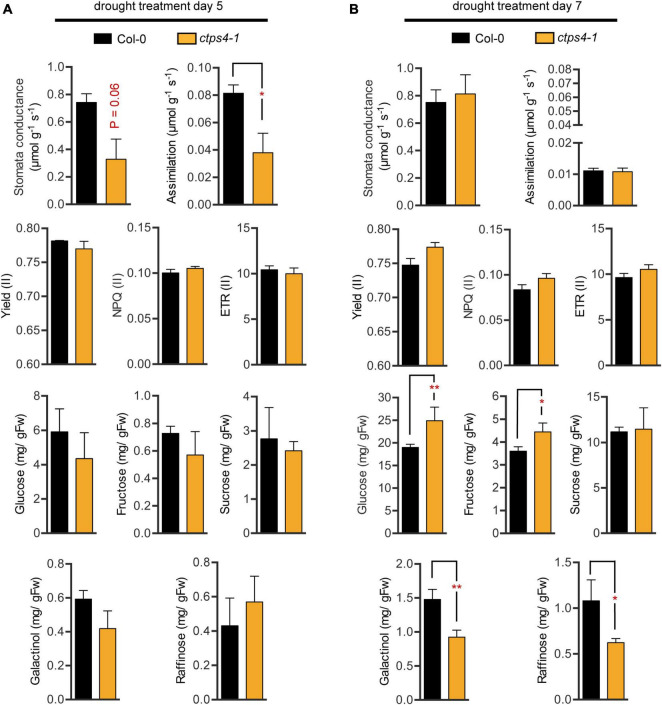
Comparison of different physiological parameters between wild type (WT) and *ctps4-1* after 5 and 7 days of pDR. Indicated parameters were determined after **(A)** 5 days pDR and **(B)** 7 days pDR. Stomata conductance, CO_2_ assimilation, and photosynthetic parameters were measured by non-invasive methods (gas exchange, fluorescence imaging). Whole rosettes were harvested and extracted for sugar quantification. Effective quantum yield (II) [Yield (II)], non-photochemical quenching [NPQ (II)], electron transport rate [ETR (II)]. Plotted are the means of *n* = 3 biological replicates ± SE. For statistical analysis, Student’s *t*-test was performed (*p < 0.05, **p < 0.01; n.s. no significance).

Water deprivation can lead to stomata closure and thus reduced CO_2_ uptake and impaired assimilation. We tested these parameters and photosynthesis parameters in addition. Since the most pronounced differences were observed after 5 and 7 days of drought, we focused on these time points. After 5 days of progressive drought, stomata conductance (*p*-value 0.06) and CO_2_ assimilation (*p* = 0.047) were reduced in *ctps4-1* (Figure 4A), but no significant change was observed after 7 days of pDR (Figure 4B). PAM chlorophyll fluorescence revealed no changes in effective quantum yield [Yield (II)], non-photochemical quenching [NPQ (II)], or the electron transport rate [ETR (II)] between WT and *ctps4-1* after 5 and 7 days of pDR (Figures 4A,B).

Sugar contents increased over three-fold from day 5 to day 7 with respect to glucose, fructose, and sucrose and over two-fold for galactinol and raffinose. During that time, fresh weight loss was around 30%; thus, plants clearly accumulated sugars during this phase of pDR. Among lines, variation in sugar contents was observed after 7 days pDR where glucose and fructose showed increased levels in *ctps41* accompanied by reductions in galactinol and raffinose (Figures 4A,B).

### *CTPS4* Expression Is Not Responsive to Abscisic Acid Treatment

**FIGURE 5:**
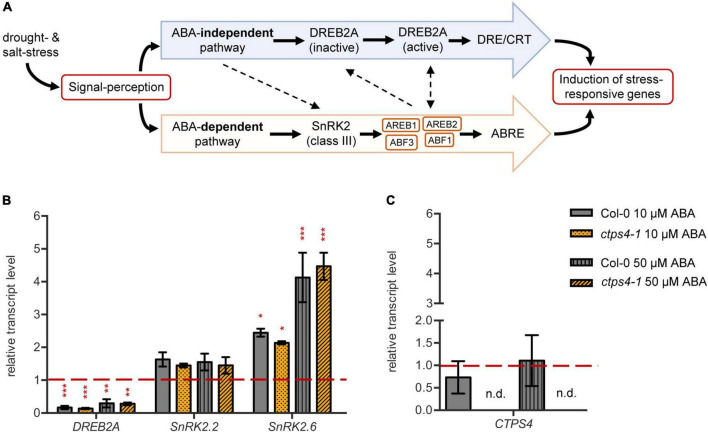
Effect of abscisic acid (ABA) treatment on drought-related genes and *CTPS4*. **(A)** Schematic representation of the ABA-dependent signaling pathway in the crosstalk with the ABA-independent signaling pathway in drought stress perception. ABA suppresses the dephosphorylation of SnRK2s (class III), which are thereby activated. The following phosphorylation of “ABA-responsive element binding proteins” and their binding factors (AREB/ABF) leads to the induction of stress-responsive genes after binding to the ABRE sequences. The ABA-independent pathway is mainly regulated by the expression of DREB2As. After post-translational modifications, they bind to DRE/CRT sequences and thereby lead to the expression of stress-responsive genes. Dashed lines are indicating putative interactions of the pathways. **(B)** Transcript levels of the drought stress regulators *DREB2A*, *SnRK2.2*, and *SnRK2.6* and **(C)** the level of *CTPS4* in leaf discs of Col-0 and *ctps4-1* after 2 h of treatment with 10 μM and 50 μM ABA, respectively, in liquid 1/2 MS medium. Expression was normalized to actin, and expression levels in leaf discs treated for 2 h with liquid 1/2 MS medium without ABA were set to 1.0. Plotted are the means of three biological replicates ± SD. For statistical analysis, one-way ANOVA was performed followed by Dunnett’s multiple comparison test (*p < 0.05, **p < 0.01, ***p < 0.001, n.d. no detection).

As *CTPS4* expression was upregulated upon drought and even more pronounced in the DROUGHT TOLERANCE SUPPRESSOR (DOR) mutant, showing a hypersensitive ABA response (Zhang et al., 2008), we investigate whether *CTPS4* expression is ABA-dependent. To do so, leaf discs from 21-day-old WT and *ctps4-1* knockout line were incubated for 2 h in 1/2 MS medium containing 0, 10, and 50 μM ABA, respectively, before the transcript levels of ABA-independent (*DREB2A*) and ABA-dependent pathway genes (*SnRK2.2* and *SnRK2.*6) and *CTPS4*, in addition, were quantified (Figure 5A). All data were normalized to *Actin2* and the control without ABA supplementation. Although the addition of ABA led to a decrease in *DREB2A* expression, the expression of genes from the ABA-dependent signaling pathways increased (Figure 5B). The most significant changes were seen in the expression of *SnRK2.6*, whereas *SnRK2.2* showed no significant changes. The addition of 10 μM ABA resulted in a doubling and 50 μM in a four-fold increase of the *SnRK2.6* transcript level. Overall, the control plants showed no significant changes in gene expression compared to the *ctps4-1* knockout plants (Figure 5B). Addition of 10 or 50 μM ABA did not lead to a change in *CTPS4* expression (Figure 5C).

### Interaction of Cytidine Triphosphate Synthase Isoforms Is Revealed by Bimolecular Fluorescence Complementation Studies

Although *CTPS4* plays a role in the drought response, it is still unclear what its exact physiological function is. Besides acting in pyrimidine *de novo* synthesis, CTPS can form cytoophidia, filamentous structures able to interact with microtubules in rice (Yoon et al., 2021) and proposed to be involved in affecting cell structure in *Caulobacter crescentus* (Ingerson-Mahar et al., 2010). Such filamentation can occur especially under stressful conditions (Noree et al., 2014; Petrovska et al., 2014; Lynch et al., 2017). Because the increasing transcript level of *CTPS4* under drought stress is still not outreaching those of other isoforms, we speculated about mechanistic interactions between isoforms.

To elucidate this issue and to determine whether the different CTPS isoforms in Arabidopsis interact with each other at all, the BiFC method was used. For this, constructs were created in which one protein carries a C-terminal YFP fragment, and the other protein carries a corresponding N-terminal YFP fragment. Transient expression was performed in *N. benthamiana* leaves.

**FIGURE 6:**
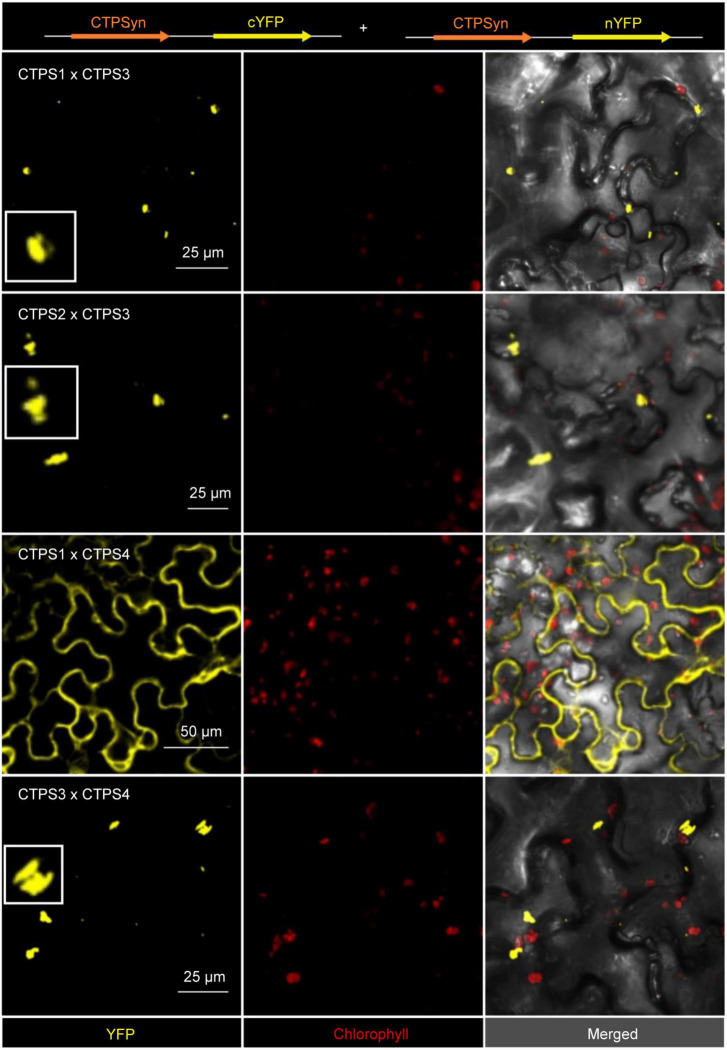
Bimolecular fluorescence complementation (BiFC) assay to determine interactions of the CTPS 1-4. Shown are the interaction of combinations of each two different isoforms after transient expression in *Nicotiana benthamiana* leaves. Zoom in on selected filaments are shown in white boxes. CTPS was fused C- or N-terminally in the vector pUB with either a C- or N-terminal YFP fragment. Confocal laser scanning was applied for imaging. CTPS, cytidine triphosphate synthase; YFP, yellow fluorescent protein.

Interactions were identified for the following combinations: (1) C-terminal-fused CTPS3 and C-terminal-fused CTPS1, these proteins showed interaction in the form of filament formation with each other (Figure 6). (2) C- and N-terminal fusions of YFP fragments to CTPS3 and CTPS2, respectively, and also resulted in filament generation. (3) CTPS4 and CTPS1 were cytosolically dissolved after the expression of C-terminal fusion constructs. (4) C-terminal fusions of CTPS4 and CTPS3 with the respective YFP fragments showed again filament formation (Figure 6). Combinations of CTPS2 with CTPS1 and CTPS2 with CTPS4 showed no interactions with each other (not shown).

## Discussion

Drought represents a severe abiotic stress factor affecting plant growth and development. It is expected that climate change more often provokes weather scenarios of too much or not enough precipitation, the latter making water a limiting resource. Therefore, an understanding of the consequences of drought stress and water use strategies is of importance for sustainable agriculture (Harb et al., 2010).

The protein family of Arabidopsis CTPS (*At*CTPS), catalyzing the final step in pyrimidine *de novo* synthesis, consists of five members (Daumann et al., 2018). Among these, At*CTPS4* in particular responds to abiotic stress. During the applied pDR treatment At*CTPS4* expression increased up to 500-fold at day 10 (Figure 1A) and up to 7.5-fold under salt stress (Figure 1B). This point in pDR is characterized by fully wilted leaves of corresponding plants. The observation of massively increased *At*CTPS4 expression under drought and salt stress conditions corresponded to similar observations in genome wide expression studies (Hruz et al., 2008; Zhang et al., 2008).

To test whether At*CTPS4* upregulation is required for drought resistance of Arabidopsis, two previously identified T-DNA insertion lines (*ctps4-1* and *ctps4-2*) (Daumann et al., 2018) were tested in our setup. We first verified our pDR setup by determining pot weights, FW/DW ratios, and the expression of marker genes. FW/DW ratios were roughly 10 for well-watered plants, before the drought and after recovery, but not significantly altered between plant lines (Figures 3D,G). During pDR, FW/DW ratio was reduced as can be expected and reached a value of 6 after 7 days of drought, further substantiating that our pDR experiment worked (Figures 3D,G).

Clearly, after 5 and 7 days of drought treatment, both mutant lines accumulated significantly less FW, and the same was true when plants recovered from both time points (Figures 3E,F). Furthermore, reduced assimilation of *ctps4-1* after 5 days of pDR was accompanied by reduced stomatal conductance (Figure 4A) pointing to reduced turgor pressure leading to stomata closure and less available CO_2_ for assimilation. After 7 days pDR, soluble sugars, including galactinol and raffinose, increased in both, mutants and WT compared to the 5-day timepoint, in line with the typical drought response. Raffinose and galactinol are of interest because they are key compatible solutes involved in the response to environmental stress (Fabregas and Fernie, 2019). However, *ctps4-1* plants exhibited higher glucose and fructose levels accompanied by lower galactinol and raffinose amounts (Figure 4B). Overall, these observations indicated to us that upregulation of *CTPS4* expression upon drought is linked to a specific function of this isoform for the plant’s drought stress resistance. The resulting questions were: (1) how is *CTPS4* expression regulated? and (2) what is the physiological function of this isoform under drought stress?

Drought-responsive genes can be under the control of ABA-dependent or -independent pathways. When leaf discs were incubated in the presence of ABA, corresponding marker-genes (*SnRK2*.2 *SnRK2*.6) were upregulated, whereas this was not observed for At*CTPS4* (Figures 5B,C). Moreover, no change in the ABA response of *SnRK2.2* or *SnRK2.6* was observed in At*CTPS4* knockout plants (Figure 5C). Therefore, we regard it as less likely that At*CTPS4* expression is controlled by or interacts with the ABA-dependent pathway. Histochemical analysis of *proCTPS4*:GUS lines revealed staining in roots and hypocotyl. Upon drought, staining became more intense, and the stained regions became enlarged. Mature, drought-treated plants showed staining of vascular tissues at the leaf base in addition (Figures 2B,C). Root functions in water uptake and together with the hypocotyl in the transport of water to the sites of photosynthesis. Although the expression profile fits to a function in the drought stress response, it cannot be excluded that At*CTPS4* expression in the leaf, even under drought stress, is too low to be detected by histochemical staining. In fact, in control tissues, At*CTPS4* is hardly expressed at all and after massive upregulation reaches levels still lower than determined for *CTPS2* and *CTPS3* (Supplementary Figure 1).

The molecular function of CTPS is the production of CTP from UTP, which marks the final step in pyrimidine *de novo* synthesis (Long and Pardee, 1967; Levitzki and Koshland, 1972), and this function is conserved in plants (Daumann et al., 2018; Yoon et al., 2021). *At*CTPS2 was identified as essential for early embryo development, chloroplast DNA synthesis, and photosynthesis (Alamdari et al., 2021; Hickl et al., 2021). This function can be explained by the demand for cytidine and deoxycytidine nucleotides for RNA and DNA synthesis, especially when growth is fast as in developing seeds and the establishment of photosynthesis in young seedlings (Bellin et al., 2021b). In line with this, CTPS1 from the rice was identified as essential for endosperm development (Yoon et al., 2021).

Levels of guanine and cytosine containing (deoxy) nucleotides are low among nucleobases and thus can become limiting factors in nucleic acid synthesis under such conditions. The two rate-limiting enzymes for cytidine and guanosine nucleotide synthesis CTPS and inosine monophosphate dehydrogenase (IMPDH) physically interact in mammalians to allow for coordinated nucleotide synthesis in filamentous structures also named cytoophidia (Liu, 2016; Chang et al., 2018). In addition, the two human CTPS isoforms exhibit different physiological functions. However, hCTPS2 is regarded as a housekeeping enzyme, whereas hCTPS1 is specifically required for lymphocyte proliferation, and both can produce cytoophidia (Martin et al., 2014). A low affinity of hCTPS1 to feedback inhibition allows the build-up of high CTP levels needed for high cell proliferation (Lynch et al., 2021). If similar adaptations of enzyme characteristics and protein interactions exist in plants is so far unclear. However, Arabidopsis CTPS isoforms 3, 4, and 5 are able to form cytoophidia too (Daumann et al., 2018; Alamdari et al., 2021) as shown for CTPS proteins from all kingdoms of life (Zhou et al., 2020). Expression of *CTPS3*, *4*, and *5* lead to the formation of large irregular filaments, and isoforms 3 and 4 colocalized after co-expression and by this affected cytoophidia size (Daumann et al., 2018; Alamdari et al., 2021). When we analyzed *At*CTPS isoform interactions in a split YFP system, we found hetero-oligomerization between *At*CTPS3 with *At*CTPS1 and *At*CTPS with *At*CTPS4 (Figure 6). All these interactions provoked the formation of punctate or rod-like structures, presumably shorter as observed when isoforms were expressed alone and in agreement with Alamdari et al. (2021). Co-expression of *At*CTPS1 and 4 produced both cytoophidia and soluble YFP signals. Thus, it is possible that the upregulated *At*CTPS4 expression would lead to increased interaction with other isoforms affecting their oligomeric (filamentation) status and possibly also their activity. Interestingly, *At*CTPS3 can force *At*CTPS1 to form mixed filaments, whereas *At*CTPS1 inhibits filament formation when interacting with *At*CTPS4. However, up to now, we do not know whether CTPS filaments in Arabidopsis, if they exist *in vivo*, are inactive, as in *Escherichia coli* (Barry et al., 2014), or active as observed for mammals (Lynch et al., 2021). While activation of CTPS activity could help counteract imbalances in (deoxy) nucleotide availability during drought progression, inactive cytoophidia could allow for a fast reestablishment of CTPS activity by depolymerization after the drought is over. Balancing nucleotide levels is crucial for organisms, especially under growth and stress conditions, and CTPS is a key regulatory element in this scenario. This statement is supported by the following observations: (1) it was shown that *Caenorhabditis elegans* shut down germ cell proliferation in response to pyrimidine deprivation, subsequently leading to altered phosphorylation of CTPS-1 (Jia et al., 2020); (2) mutant phenotypes in plants lacking CTPS2 could be rescued by feeding with deoxycytidine indicating that this metabolite was limiting (Alamdari et al., 2021; Bellin et al., 2021b); and (3) upon growth signals, nucleotide *de novo* synthesis is upregulated by TOR and CTPS is one of the targets (Busche et al., 2021). An effect of osmolarity on CTPS filament fragmentation was observed in *Saccharomyces cerevisiae*, supporting the role of cytoophidia in stress resistance (Li and Liu, 2021).

Drought is often accompanied by the accumulation of ROS (Mukarram et al., 2021), which can lead to DNA damage and further to altered cell cycle regulation. A direct link between salinity and drought stress and deregulation of the cell cycle, resulting in a low cell proliferation rate was established in maize (Kamal et al., 2021).

Important roles of individual plant CTPS isoforms for DNA synthesis in young seedlings and embryo development in Arabidopsis and rice endosperm development have been identified (Alamdari et al., 2021; Bellin et al., 2021b; Hickl et al., 2021; Yoon et al., 2021). Thus, one could imagine a scenario where *At*CTPS4 expression upon stress acts in concert with other CTPS isoforms to attenuate negative consequences of ROS-induced DNA damage or to allow sufficiently high synthesis of (deoxy) CTP. In line with this, observed defects in the nuclear division in *OsCTPS1* mutants support the idea of multiple isoforms acting together to secure rapid nuclear division (Yoon et al., 2021).

Filament formation by CTPS can exhibit secondary functions apart from regulating enzyme activity. For example, CTPS filaments from *C. crescentus* regulate cell curvature by interacting with the cytoskeletal filament crescentin (Ingerson-Mahar et al., 2010). Moreover, the catalytic and morphogenic functions could be complemented by the expression of CTPS from *E. coli*, pointing toward the conservation of both functions. An association of CTPS with the cytoskeleton in *Drosophila melanogaster* follicle cells was found (Liu, 2010), and recently microtubule interaction with *Os*CTPS1 was shown (Yoon et al., 2021). Maybe, filament forming metabolic enzymes like CTPS contribute to cell stabilization when turgor pressure is impaired as it is the case under drought.

## Accession Numbers

CTP synthase 1 (AtCTPS1; At1g30820); CTP synthase 2 (AtCTPS2; At3g12670); CTP-synthase 3 (AtCTPS3; At4g02120); CTP synthase 4 (AtCTPS4; At4g20320); and Actin 2 (Act2; At3g18780).

## Data Availability Statement

The original contributions presented in the study are included in the article/Supplementary Material, further inquiries can be directed to the corresponding author.

## Author Contributions

DH, MK, and TM designed the research. DH, MK, and ED performed the experiments. DH, MK, ED, VS, and LB analyzed the data. DH and MK created and screened the transgenic plants. LB, VS, ED, and TM wrote the manuscript with contributions and approval from all authors.

## Conflict of Interest

The authors declare that the research was conducted in the absence of any commercial or financial relationships that could be construed as a potential conflict of interest.

## Publisher’s Note

All claims expressed in this article are solely those of the authors and do not necessarily represent those of their affiliated organizations, or those of the publisher, the editors and the reviewers. Any product that may be evaluated in this article, or claim that may be made by its manufacturer, is not guaranteed or endorsed by the publisher.
