# Supplementary material for: Cytidine Triphosphate Synthase Four From Arabidopsis thaliana Attenuates Drought Stress Effects
Source: Front Plant Sci. 2022 Mar 10;13:842156. doi: 10.3389/fpls.2022.842156 (PMC8960734; doi:10.3389/fpls.2022.842156)
Supplement: Supplementary file 1 [file Data_Sheet_1.pdf]

Supplementary Figures

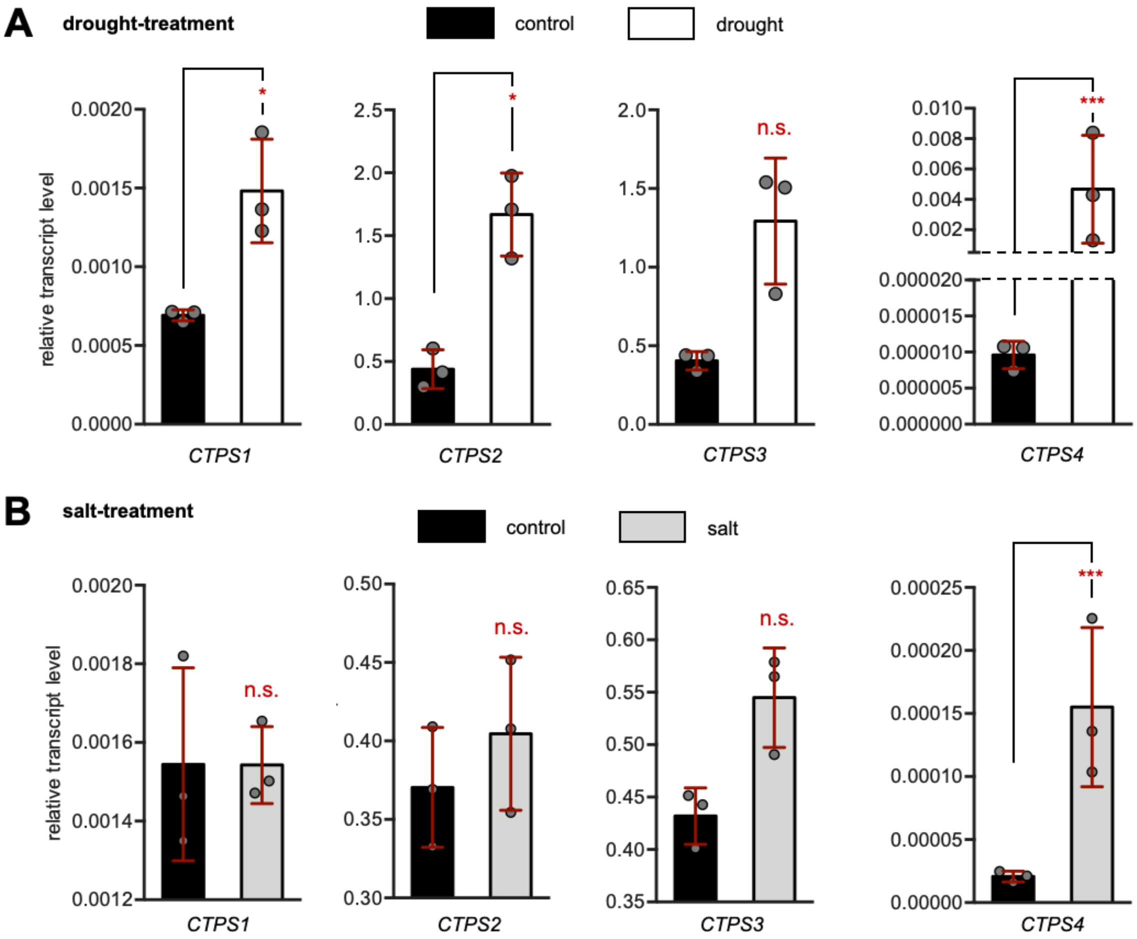

Supplementary Figure 1. CTPS4 expression is highly upregulated upon drought and salt stress. (A) Relative transcript levels of *CTPS1-4* in wild type plants after 10 days of drought treatment. Expression was normalized to *Actin2*. (**B**) Analysis of relative transcript levels of *CTPS1-4* in wild type plants grown on soil and watered with 150 mM NaCl to induce salt stress. Expression was normalized to *Actin2*. Plotted are the means of biological replicates +/- SD. For statistical analysis, one-way ANOVA was performed followed by Dunett's multiple comparison test (* p< 0.05, *** p<0.001, n.s. no significance)

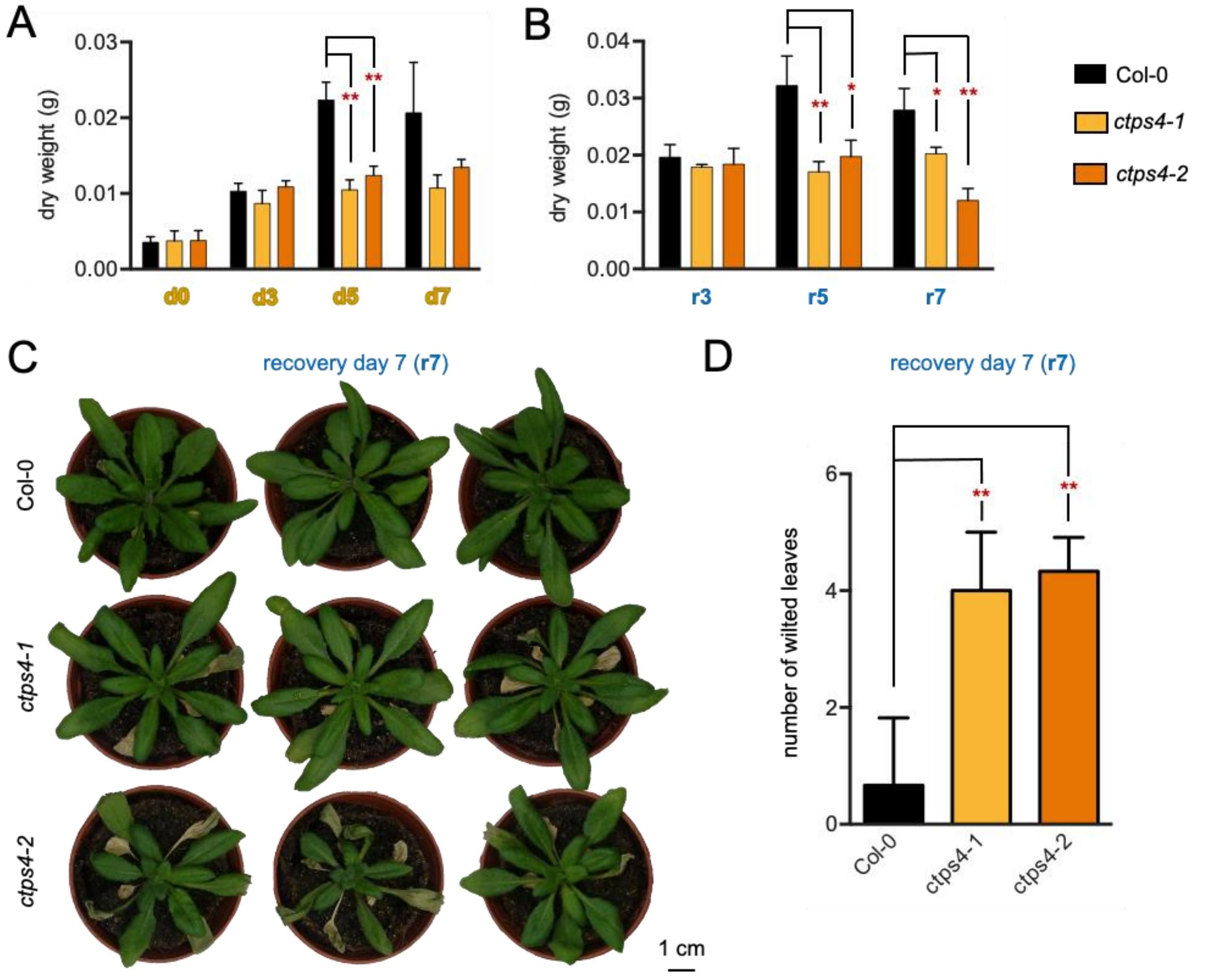

Supplementary Figure 2. Effects of progressive drought stress on CTPS4 knock-out plants. Plants were grown according to the regime used throughout this work as shown in **Figure 3 (A, B)** Dry weight of harvested complete rosettes after **(A)** 3, 5 and 7 days of drought or **(B)** three days of recovery from the same time points (r3, r5 and r7) (**C**, **D**) Images of recovered plants and number of wilted leaves after seven days of drought followed by three days of recovery (r7). Plotted are the means of n = 3 biological replicates +/- SD. For statistical analysis, one-way ANOVA was performed followed by Dunett's multiple comparison test (* $p< 0.05$, ** $p<0.01$).

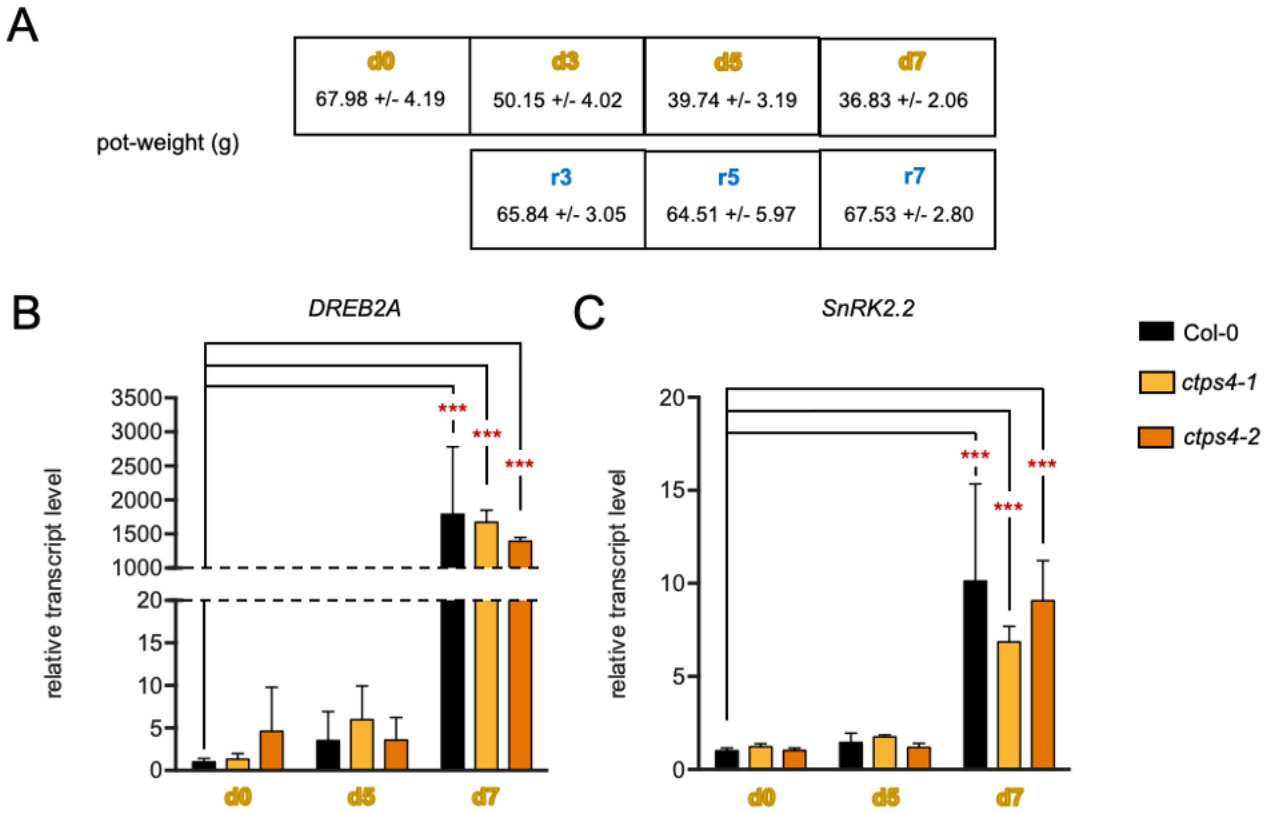

Supplementary Figure 3. Water loss of cultivation pots and accompanied induction of marker gene expression during pDr. Plants were grown according to the regime used throughout this work as shown in **Figure 3** Letters “d” and “r” followed by a number indicate days of drought or recovery from days of drought (**A**) pot weight was determined after removal of plants. (n = 9). (**B, C**) Expression of drought markers **(B)** *DREB2A* and (**C**) *SnRK2.2* after indicated times. Expression at d0 was set to 1 after normalization to *Actin2*. Plotted are the means of n = 3 biological replicates +/- SD. For statistical analysis, one-way ANOVA was performed followed by Dunett’s multiple comparison test (*** $p<0.001$).
