# Supplementary material for: Cytidine Triphosphate Synthase Four From Arabidopsis thaliana Attenuates Drought Stress Effects
Source: Front Plant Sci. 2022 Mar 10;13:842156. doi: 10.3389/fpls.2022.842156 (PMC8960734; doi:10.3389/fpls.2022.842156)
Supplement: Supplementary file 2 [file Data_Sheet_2.PDF]

Supplementary Tables

Supplementary Table 1. List of Primers used in this work.

**Primers for cloning**	
**Reporter-GUS construct**	
CTPS4 Promotor_fwd	ACTCTAGCGTTTGTAATGATATC
CTPS4 Promotor_rev	CTTCTTCCTTTTGTTTCCTTAAAC
attB_*CTPS4* Promotor_fwd	GGGGACAAGTTTGTACAAAAAAGCAGGCTTCACTCTAG CGTTTGTAATGATATC
attB_*CTPS4* Promotor_rev	GGGGACCACTTTGTACAAGAAAGCTGGGTCCTTCTTCC TTTTGTTTCCTTAAAC
**qRT-PCR Primer**	
Actin_fwd	CTTGCACCAAGCAGCATGAA
Actin_rev	CCGATCCAGACACTGTACTTCCTT
CTPS1_fwd	GCATCGTCACAGATACGAGGTG
CTPS1_rev	CCATGCGTTTGCCAGTTTCATC
CTPS2_fwd	TGCAAGTCTGCCAAGCTGTACG
CTPS2_rev	ATCTGTGCCTGTGTCGCTCATC
CTPS3_fwd	ACAGGGAAACGTGTTGAGGTG
CTPS3_rev	GCTTCCTCGCAGCCAATATAAACC
CTPS4_fwd	TGCAAAGGAGCCTGCATTAGAGG
CTPS4_rev	TCCCAACCACAGCAATTCTTACCG
DREB2A fwd	CAGTGTTGCCAACGGTTCAT
DREB2A rev	AAACGGAGGTATTCCGTAGTTGAG
SnRK2.2 fwd	TCGACAGGAATATGATGGCAAGC
SnRK2.2 rev	CGATAATCTCGTGGCTCCTGTG
SnRK2.6 fwd	TGTTGCTGACCCTGCAAAGAGG
SnRK2.6 rev	TATGCTTTGGCCCGGTTGATCC

**Primer for interaction studies**	
CTPS1_cDNA_fwd	ATGAAGTACGTGCTTGTAACAG
CTPS1_ cDNA_rev	CTATCTAGTGTAGAGGCCATT
CTPS2_ cDNA_fwd	ATGAAGTACGTTTTGGTGACA
CTPS2_ cDNA_rev	TCAGTGGTGAAGCCCGTTT
CTPS3_ cDNA_fwd	ATGAAGTACGTATTGGTGACT
CTPS3_ cDNA_rev	TCAATTGCTTAAATGGGCTTGA
CTPS4_ cDNA_fwd	ATGAAGTATGTGGTTGTTTCA
CTPS4_ cDNA_rev	TCACGAGTAGACACGATCACA
CTPS_attb1 adapter	GGGGACAAGTTTGTACAAAAAAGCAGGCT
CTPS_attb2 adapter	GGGGACCACTTTGTACAAGAAAGCTGGGT
CTPS1_attb insert_fwd	AAAAAGCAGGCTTAATGAAGTACGTGCTTGTAACAGGAG
CTPS1_attb insert_rev	AGAAAGCTGGGTTTCTAGTGTAGAGGCCATTGCAGT
CTPS2_attb insert_fwd	AAAAAGCAGGCTTAATGAAGTACGTTTTGGTGACAGGAG
CTPS2_attb insert_rev	AGAAAGCTGGGTTGTGGTGAAGCCCGTTTCCAT
CTPS3_attb insert_fwd	AAAAAGCAGGCTTAATGAAGTACGTATTGGTGACTGGAGG
CTPS3_attb insert_rev	AGAAAGCTGGGTTATTGCTTAAATGGGCTTGAAGAAGCT
CTPS4_attb insert_fwd	AAAAAGCAGGCTTAATGAAGTATGTGGTTGTTTCAGGAGG
CTPS4_attb insert_rev	AGAAAGCTGGGTTCGAGTAGACACGATCACATAAACTGTACAC
